# Discovery of Neuroprotective Agents Based on a 5-(4-Pyridinyl)-1,2,4-triazole Scaffold

**DOI:** 10.1021/acschemneuro.1c00849

**Published:** 2022-02-18

**Authors:** Rosaria Gitto, Serena Vittorio, Federica Bucolo, Samuel Peña-Díaz, Rosalba Siracusa, Salvatore Cuzzocrea, Salvador Ventura, Rosanna Di Paola, Laura De Luca

**Affiliations:** † Department of Chemical, Biological, Pharmaceutical and Environmental Sciences, University of Messina, Viale F. Stagno D’Alcontres 31, I-98125 Messina, Italy; ‡ Institut de Biotecnologia i Biomedicina, Universitat Autonoma de Barcelona, 08193 Bellaterra, Spain; § Departament de Bioquimica i Biologia Molecular, Universitat Autonoma de Barcelona, 08193 Bellaterra, Spain; ∥ ICREA, Passeig Lluis Companys 23, 08010 Barcelona, Spain

**Keywords:** 5-(4-pyridinyl)-1,2,4-triazoles, synthesis, Parkinson’s disease, alpha synuclein, MPTP

## Abstract

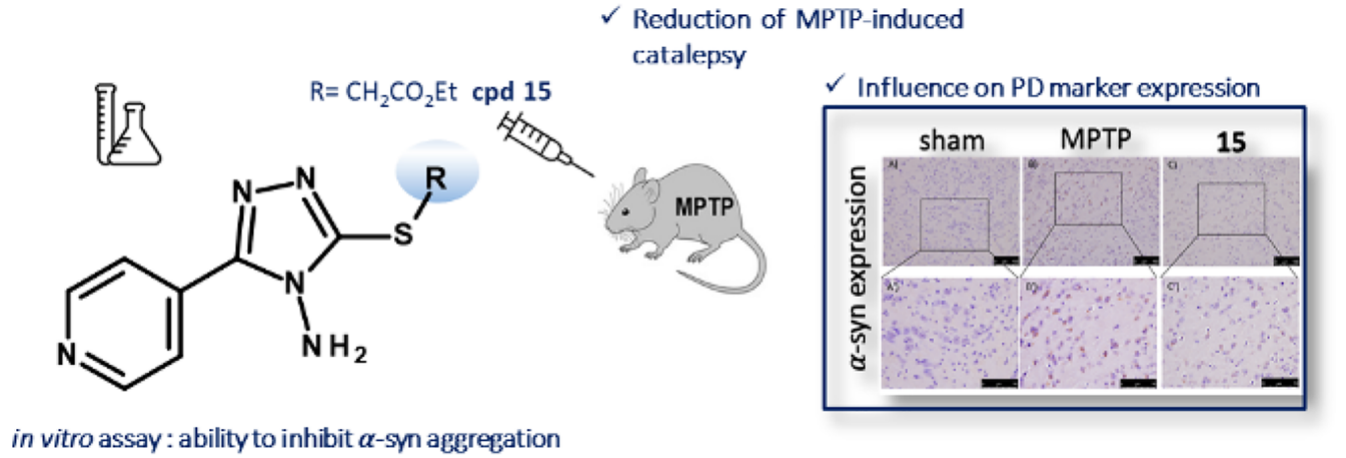

Parkinson’s disease (PD) is characterized by the death of dopaminergic neurons. The common histopathological hallmark in PD patients is the formation of intracellular proteinaceous accumulations. The main constituent of these inclusions is alpha-synuclein (α-syn), an intrinsically disordered protein that in pathological conditions creates amyloid aggregates that lead to neurotoxicity and neurodegeneration. The main goal of our study was to optimize our previously identified α-syn aggregation inhibitors of 5-(4-pyridinyl)-1,2,4-triazole chemotype in terms of in vivo efficacy. Our efforts resulted in the identification of ethyl 2-((4-amino-5-(pyridin-4-yl)-4*H*-1,2,4-triazol-3-yl)thio)acetate (**15**), which displayed the ability to prevent 1-methyl-4-phenyl-1,2,3,6-tetrahydropiridine-induced bradykinesia as well as to affect the levels of PD markers after the administration of the same neurotoxin. In addition to the in vivo evaluation, for the 5-(4-pyridinyl)-1,2,4-triazole-based compounds, we measured the prevention of the fibrillization process using light scattering and a ThT binding assay; these compounds have been shown to slightly reduce the α-syn aggregation.

## Introduction

A progressive loss of dopaminergic neurons in the *substantia nigra pars compacta* of the brain characterizes Parkinson’s Disease (PD).^1^ Thus, the neurodegeneration results in a significant reduction of dopamine (DA) in the synaptic terminals of the dorso-striatum, so that PD has recently been considered as a synaptopathy. Although there is still much debate about the main cause of PD, the analysis of the brain of PD patients revealed a common factor: the formation of intraneuronal inclusions, named Lewy bodies and Lewy neurites, formed by aggregates of a disordered protein called alpha synuclein (α-syn). α-Syn is a small protein localized in the presynaptic terminal,^2,3^ whose sequence could be dissected in three distinctive domains: the N-terminal domain, central domain NAC, and C-terminal domain.^4^ α-Syn is physiologically found as a soluble monomer, but after interaction with phospholipids it adopts an α-helical structure.^5^ In the misfolded state, the aggregation of α-syn into amyloid fibrils leads to neuronal pathological inclusions located both in the neuron soma and in axons.^6,7^ It is well-known that this process causes cytotoxicity through different mechanisms such as the increase of lipid membrane permeabilization, the mitochondrial damage, and the oxidative stress.^8^ Moreover, the misfolded extracellular α-syn establishes a prion-like mechanism of propagation from neurons to glial cells.^9^ Finally, the misfolded α-syn acts as an antigen, thus triggering an immune response closely associated with neuroinflammatory events.^10^

**Figure 1:**
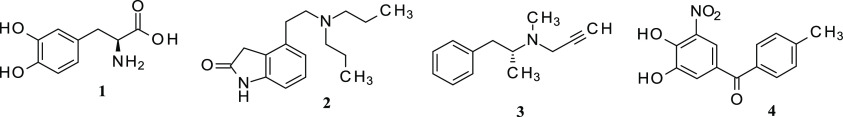
Chemical structures of well-known therapeutics for PD.

The therapeutic treatment is exclusively focused on alleviating motor symptoms so that the restoring of DA levels represents the widespread approach by using the prodrug L-Dopa **1** (Figure 1), which crosses the blood-brain barrier (BBB) and is converted to DA.^1,11^ There are also therapeutics (see Figure 1) that target the dopamine receptor (e.g., ropinirole, **2**) or inhibit monoamine oxidase B (MAO B) (e.g., selegiline, **3**) and catechol-*O*-methyltransferase (COMT) (e.g., tolcapone, **4**).^12,13^ Sadly, the side effects of the above-mentioned therapeutics have been turned to the exploration of new targets.

On the basis of these considerations, the inhibition of α-syn aggregation might be considered a valuable approach to research new agents that might be able to prevent the disease progression in place of relieving the symptoms.

**Figure 2:**
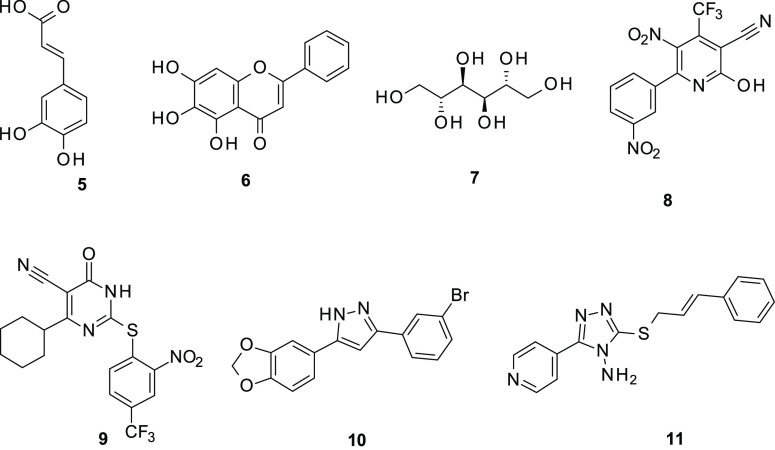
Small molecules as α-syn aggregation inhibitors.

Recently, it has been suggested that a therapeutic strategy might be achieved inhibiting or reversing the α-syn aggregation by using peptides or small molecule able to interact with α-syn protein through distinct modalities and to prevent the formation of oligomers and subsequently amyloid fibrils.^14^ Currently two main classes of small molecules have been identified through in vivo screening assays; they are polyphenol and nonpolyphenol compounds (see Figure 2) from natural and synthetic sources. Among polyphenol compounds, it has been demonstrated that caffeic acid (**5**) possesses an antifibrillating ability in a dose-dependent manner against the aggregation of α-syn induced by treatment with escitalopram. Indeed, by analyzing the structure of phenolic compounds, it was observed that the presence of the catechol moiety leads to an antiamyloidogenic activity.^15^ The flavone baicalein (**6**) proved to prevent the α-syn cytotoxicity and inhibit and stabilize the oligomerization of α-syn, thus blocking the fibril formation. Concerning nonpolyphenol derivatives, inhibitory effects on the aggregation of α-syn were found for mannitol (**7**) and several terpenoids as well as alkaloids.^16,17^ Collecting structure-affinity relationship information, it was observed that the presence of aromatic rings and/or planar structures could represent an important requirement for inhibitory activity.^18^

A number of promising small molecule inhibitors from synthetic sources have been discovered using innovative approaches, including high-throughput screening. SynuClean-D (**8**), ZPD-2 (**9**), and anle318b (**10**) were demonstrated to inhibit the α-syn aggregation and to prevent their propagation in PD.^19−21^

On the basis of the knowledge that the aggregation-prone NAC domain of α-syn is crucial for the conformational shift of the protein to β-sheets, potential α-syn aggregation inhibitors were developed as ligands of the target residues 69–72. Accordingly, we focused our attention to identify of newer α-syn aggregation inhibitors oriented toward the NAC domain of α-syn.^22^ From a set of 4-amino-5–(4-pyridinyl)-4*H*-1,2,4-triazole-derived compounds, we identified the 3-(cinnamylsulfanyl)-5-(4-pyridinyl)-1,2,4-triazol-4-amine (**11**) that proved to reduce α-syn aggregation in an in vitro assay. Docking studies suggested the binding into a specific site placed between N-terminal and NAC domains. As a continuation of these promising achievements, we now report the exploitation of a pyridinyl-triazole scaffold of hit compound **11** for further in vivo and in vitro investigations. Therefore, the new designed compounds were screened as α-syn aggregation inhibitors. Moreover, we performed in vivo studies by means of the experimental protocol of 1-methyl-4-phenyl-1,2,3,6-tetrahydropiridine (MPTP)-induced degeneration of dopaminergic neurons.

## Result and Discussion

### Design and Synthesis of New 4-Amino-5-(4-pyridinyl)-4H-1,2,4-triazole-Derived Compounds

**Figure 3:**
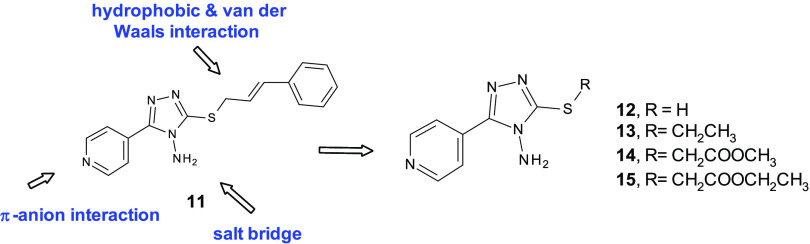
Newly studied compounds **12**–**15** bearing the 5-(4-pyridinyl)-1,2,4-triazol-4-amine core.

Our proposed pharmacophore for α-syn aggregation inhibition consisted of (i) a salt bridge contact, (ii) an unusual π-anion interaction, and (iii) several hydrophobic and van der Waals interactions (see Figure 3). On the basis of this pharmacophore pattern, we designed new compounds with the aim to achieve further structural data for this series of compounds. Specifically, we chose to maintain the pyridinyl-triazol-4-amine core and explored the eastern region of prototype **11** by the removal of the cinnamyl fragment and the insertion of small fragments. In detail, the new compounds were prepared to study the effect of introducing small substituents able to increase H-bond donor/acceptor contacts as well as hydrophobic interactions in the subpocket lined by crucial NAC domain residues. Therefore, we investigated the α-syn aggregation inhibitory effects of the starting material 4-amino-5-(4-pyridinyl)-4*H*-1,2,4-triazole-3-thiol (**12**) and its corresponding three derived analogue compounds **13**–**15** reported in Figure 3.

The synthetic route for compounds **13**–**15** was based on the well-established reaction of the starting material **12** with an opportune alkyl halide in alkaline medium as previously reported by us.^22^ The synthesized compounds were structurally characterized by means of spectroscopic measurements, and all data are reported in the Methods section.

### In Vivo Studies

Among the new synthesized compounds, we selected ethyl 2-((4-amino-5-(pyridin-4-yl)-4*H*-1,2,4-triazol-3-yl)thio)acetate (**15**) to ascertain its protective effect by measuring specific markers of PD. We measured the capacity of compound **15** to prevent the neurodegeneration produced by the neurotoxin MPTP investigating the levels of tyrosine hydroxylase (TH) and α-syn in the midbrain.

**Figure 4:**
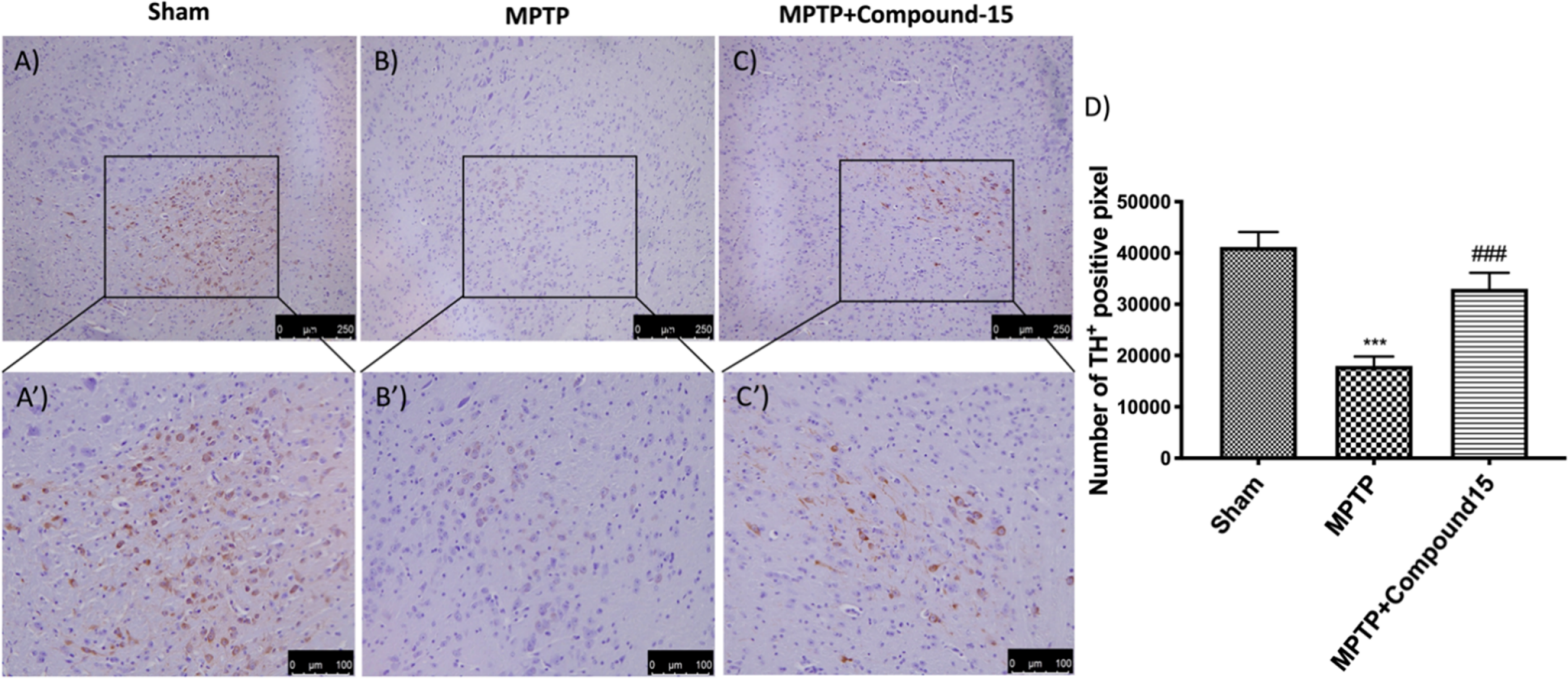
Effects of **15** on TH expression in the midbrain of MPTP-treated mice. The immunohistochemical analysis demonstrated, compared with the Sham mice (A,A′), a pronounced loss of TH-positive cells (B,B′). Animals treated with **15** showed an increase in TH expression (C,C′). Data are expressed as the percentage of TH-positive pixels and are the means ± SEM of five mice per group. (D) ***p < 0.001 vs Sham; ###*p* < 0.001 vs MPTP.

By an immunohistochemical analysis, the expression of TH-positive neurons was significantly reduced 8 d after MPTP administration (Figure 4B,B′,D) compared to the results of the Sham group (Figure 4A,A′,D). Treatment with compound **15** incremented the levels of this protein (Figure 4C,C′,D).

**Figure 5:**
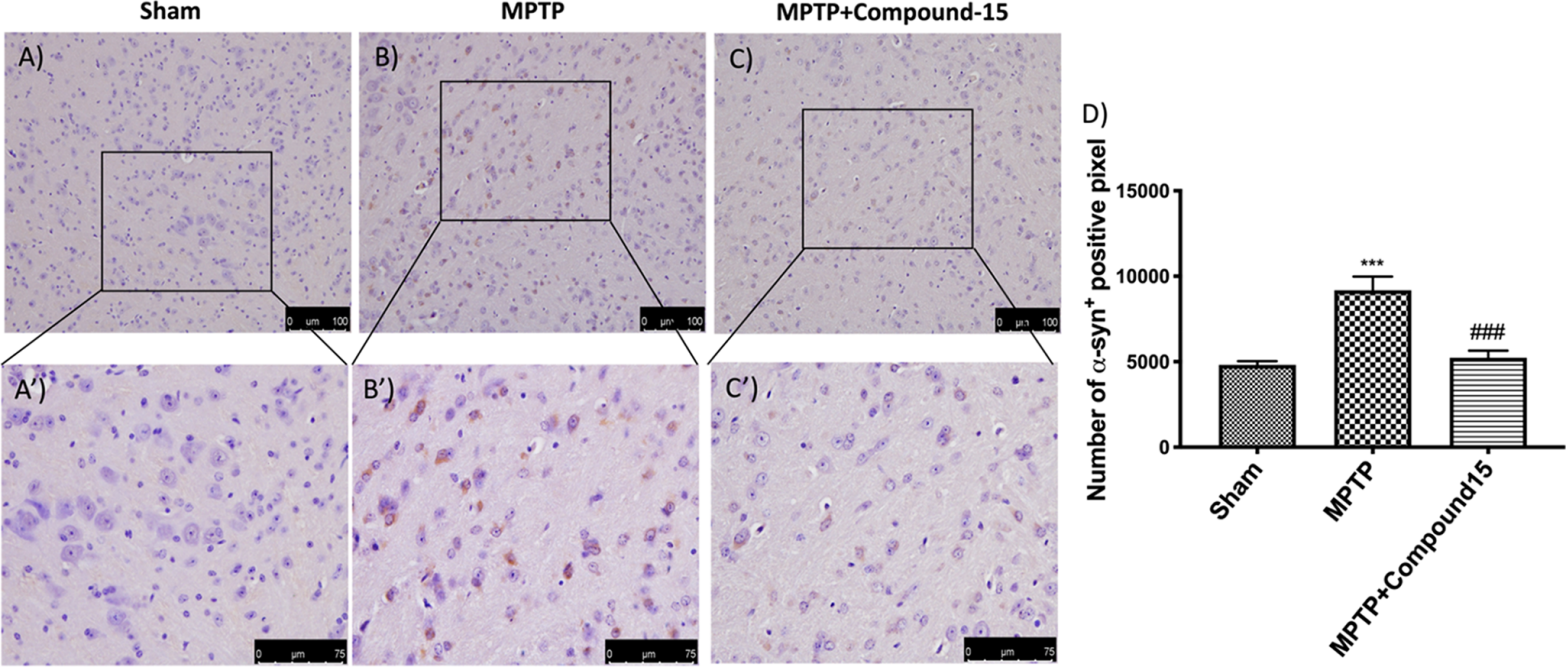
Effects of compound **15** on α-syn expression in midbrain of MPTP-treated mice. The immunohistochemical investigation discovered, compared with Sham animals (A,A′), a positive stain for α-syn (B,B′). Compound **15** treatment significantly reduced the positive stain for α-syn in the SN (C,C′). (D) ***p < 0.001 vs Sham; ###*p* < 0.001 vs MPTP. Data are expressed as the percentage of α-syn-positive pixels and are the mean ± SEM of five mice per group.

In reverse, we detected an important immunoreactivity in MPTP-damaged mice (Figure 5B,B′,D) compared with Sham animals (Figure 5A,A′,D). Rather, the compound **15** treatment appreciably reduced α-syn expression in the midbrain after MPTP intoxication (Figure 5C,C′,D).

### In Vitro Studies

**Figure 6:**
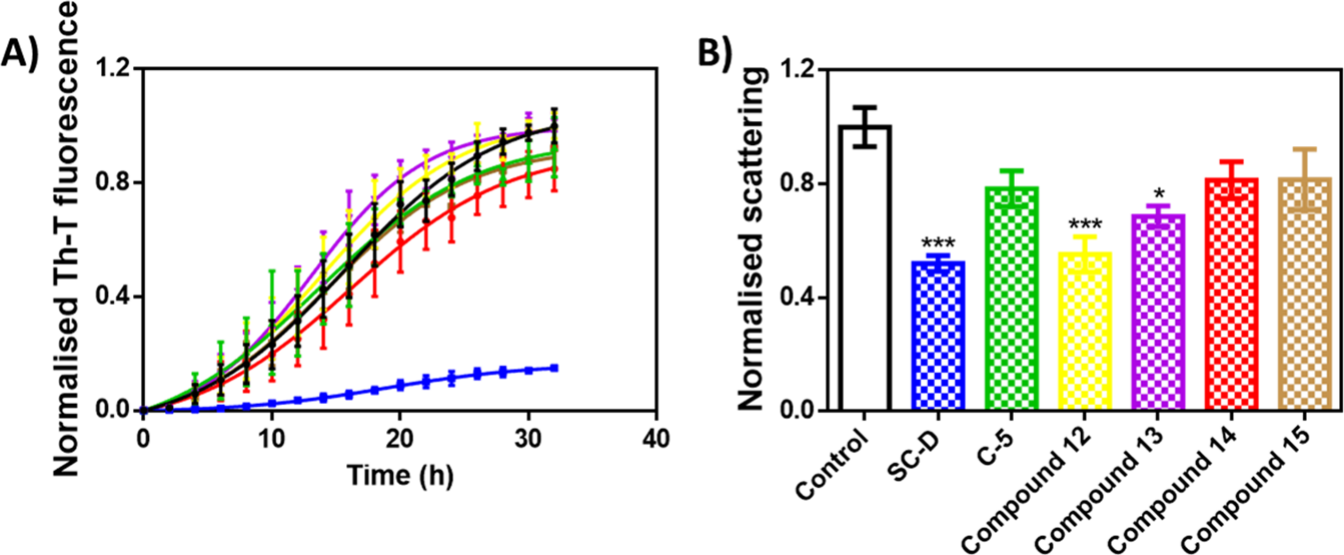
In vitro analysis of the capacity of the different compounds to inhibit α-syn aggregation. (A) Aggregation kinetics of α-syn in the absence (black) or presence of 100 μM of **12** (yellow), **13** (violet), **14** (red), or **15** (brown) compared to previously described molecules SC-D (blue) and C-5 (green). Normalized intensity of Th-T fluorescence is plotted as a function of time. (B) Light-scattering measurements at 300 nm of end-point aggregates in the absence (black) or presence of 100 μM of **12** (yellow), **13** (violet), **14** (red), or **15** (brown) compared to previously described molecules SC-D (blue) and C-5 (green). Th-T fluorescence and light-scattering is plotted as normalized means; error bars are represented as the SE of mean values. *p < 0.05 and ***p < 0.001.

On the basis of the promising in vivo effects measured for compound **15**, we moved our attention to the evaluation of the ability to reduce α-syn aggregation in vitro. Then, the four pyridinyl-triazole derivatives **12**–**15** were tested by the same protocol used for the identification of potent α-syn aggregation inhibitors like SynuClean-D (SC-D) that was used as a reference molecule together the previously reported^22^ parent compound C-5 in which a benzyl moiety (R = CH_2_Ph) is linked to a sulfur atom of compound **12**. We monitored the kinetics of aggregation of 70 μM α-syn in the absence or presence of 100 μM of the studied derivatives by following the increase in thioflavin-T (Th-T) fluorescence (Figure 6A). Compounds **12** and **13** slightly accelerated the reaction, whereas the inhibitory effect of compound **15** was indistinguishable from C-5 (8% reduction in Th-T signal), and compound **14** performed slightly better (15% reduction in Th-T signal). Light-scattering measurements at 300 nm at the end of the reaction indicated that **12**, **13**, **14**, and **15** reduced the aggregated α-syn levels in the solution (Figure 6B), with decrements of 44%, 31%, 18%, and 18%, respectively. The impacts of compounds **14** and **15** are similar to that of C-5 (21%).^22^ The discrepancy between the influence of compounds **12** and **13** in Th-T and light-scattering signals suggests that the aggregates formed in the presence of these molecules exhibit a higher affinity for Th-T, likely being richer in intermolecular β-sheet.

Overall, these studies indicated that the 5-(4-pyridinyl)-4*H*-1,2,4-triazole core could be a new chemical template to design neuroprotective agents in PD. In detail, the immunohistochemical assays revealed that compound **15** proved to enhance levels of TH and DAT in the midbrain of mice treated with neurotoxin MPTP; further, compound **15** was able to reduce the α-syn expression in the same test. Moreover, compound **15** ameliorated a motor deficit in MPTP-treated mice (see DAT and the behavioral analysis in the Supporting Information). To provide evidence for the role of α-syn aggregation inhibition in mediating the in vivo effects, we performed in vitro studies that revealed that the studied 5-(4-pyridinyl)-4*H*-1,2,4-triazole-based compounds might affect fibrillization. Taken together, these data might indicate a plausible association between the α-syn aggregation inhibition and the in vivo effects toward MPTP-induced toxicity in mice.

## Methods

### Chemistry

Reagents and solvents were purchased from commercial suppliers (Merck KGaA and Thermofisher Scientific Inc.) and were used without further purification. Melting points of synthesized compounds were recorded on a Buchi B-545 apparatus (BUCHI Labortechnik AG) and are uncorrected. The purity of compounds was evaluated by combustion analysis measurements (C, H, N) on a Carlo Erba Elemental Analyzer (Model 1106); the collected data confirmed a purity of at least 95%. A thin-layer chromatography (TLC) analysis was performed on fluorescent silica gel 60 F254 and visualized using UV light (λ = 254 nm/366 nm) or staining with iodine vapor. All ^1^H and ^13^C NMR spectra were recorded in deuterated dimethyl sulfoxide (DMSO-*d*_6_) with a Varian Gemini 500 spectrometer (Varian Inc.). The chemical shifts are given in δ (ppm), and coupling constants (*J*) are given in hertz (Hz). The exchangeable proton atoms were detected by D_2_O.

#### General Procedure for the Synthesis of Pyridinyl-Triazole Derivatives (**13**–**15**)

The starting material 4-amino-5-(4-pyridinyl)-4*H*-1,2,4-triazole-3-thiol (**12**) (1 equiv) was dissolved in MeOH (5 mL) and NaOH (1 equiv). Then, the suitable alkyl bromide derivative (1 equiv) was added to the mixture, and it was stirred at room temperature. After the mixture was stirred for 120 min, the resulting crude product was filtered, dried, and recrystallized from EtOH to give desired final derivatives **13**–**15**. The CAS numbers for **13**–**15** have been already assigned as reported below for each compound in the Supporting Information. The structural characterization as well as the physicochemical properties were generally in agreement with previous data.^23−25^

### In Vivo Studies

We performed experiments on neurodegenerative diseases with compound **15** at a dose of 10 mg/kg after a preliminary dose–response study conducted in our laboratories^26,27^ as described in more detail in the Supporting Information.

#### Experimental Groups

The mice were arbitrarily allocated into four groups:

Group 1. Sham+Veh = Vehicle solution (saline) was administered intraperitoneal during the first day, like the MPTP protocol (*N* = 10).

Group 2. Sham+**15** = Same as the Sham+Veh group, but **15** (10 mg/kg body weight, soluble in saline, orally) was administered starting 24 h after the first vehicle solution injection and continuing through seven additional days after the last administration of saline (*N* = 10).

Group 3. MPTP+Veh = MPTP solution was administered as described for the administration of saline (*N* = 10).

Group 4. MPTP+**15** = Same as the MPTP+Veh group, but **15** (at a dose of 10 mg/kg body weight, orally) was administered starting 24 h after the first MPTP administration and continuing through seven additional days after the last injection of MPTP (*N* = 10).

#### Immunohistochemical Localization of Tyrosine Hydroxylase (TH) and α-Synuclein

We used the immunohistochemical techniques as previously described.^28^ The antibodies that were incubated overnight on the brain sections were anti-TH (Millipore, 1:500 in phosphate-buffered saline (PBS), v/v, AB152) and anti-α-syn (Santa Cruz Biotechnology, 1:50 in PBS, v/v, LB509 sc-58480). To demonstrate the specificity of the antibodies, the brain sections of five mice for each group were treated either with a primary or only with a secondary antibody. The images were captured by a Zeiss microscope and Axio Vision software. The ImageJ IHC profiler plug-in was used for a densitometric analysis. When this is selected, it automatically traces a histogram profile of the deconstructed diaminobenzidine image showing a corresponding score log. The histogram profile corresponds to the positive pixel intensity value obtained from the computer software. Immunohistochemical analyses were performed by experienced people who did not know the treatment.

### In Vitro Studies

We performed α-synuclein aggregation and inhibition in vitro assays. Human wt α-syn was expressed and purified as previously indicated^29^ and kept lyophilized at −80 °C until use. Before use, the protein was gently resuspended in sterile PBS 1X and filtered through a 0.22 μm membrane to remove small aggregates. The inhibitory capacity of the compounds was assessed as previously described.^20,22,29,30^ Briefly, 70 μM of soluble α-syn was placed in a sealed 96-well plate containing 40 μM Th-T in PBS 1X, a 1/8 in. diameter Teflon polyball (Polysciences Europe GmbH) and 100 μM of the different molecules or DMSO as control samples. Each well contained a final volume of 150 μL. Samples were then incubated at 37 °C and 100 rpm in an orbital shaker Max-Q 4000 (ThermoScientific). Th-T fluorescence emission was measured every 2 h in a TECAN SPARK-1 plate reader (Tecan Trading AG) by exciting through a 430–450 nm filter and collecting with a 480–510 nm filter. Light-scattering measurements were performed in a Cary Eclipse Fluorescence Spectrophotometer (Agilent). 80 μL of end-point aggregates was inserted into a quartz cuvette and excited at 300 nm; the 90° emission was collected between 280 and 320 nm.

## Supporting Information Available

^1^H NMR spectra and physicochemical properties for synthesized compounds **13–15**. Supplementary data of in vivo studies (PDF)

## References

[ref1] Chakraborty A.; Brauer S.; Diwan A. Possible therapies of Parkinson’s disease: A review. J. Clin. Neurosci. 2020, 75, 1–4. 10.1016/j.jocn.2020.03.024. 32247740

[ref2] Cardinale A.; Calabrese V.; de Iure A.; Picconi B. Alpha-Synuclein as a Prominent Actor in the Inflammatory Synaptopathy of Parkinson’s Disease. Int. J. Mol. Sci. 2021, 22 (12), 6517 10.3390/ijms22126517. 34204581 PMC8234932

[ref3] Save S. S.; Rachineni K.; Hosur R. V.; Choudhary S. Natural compound safranal driven inhibition and dis-aggregation of α-synuclein fibrils. Int. J. Biol. Macromol. 2019, 141, 585–595. 10.1016/j.ijbiomac.2019.09.053. 31505208

[ref4] Savitt D.; Jankovic J. Targeting α-Synuclein in Parkinson’s Disease: Progress Towards the Development of Disease-Modifying Therapeutics. Drugs 2019, 79 (8), 797–810. 10.1007/s40265-019-01104-1. 30982161

[ref5] Afitska K.; Priss A.; Yushchenko D. A.; Shvadchak V. V. Structural Optimization of Inhibitors of α-Synuclein Fibril Growth: Affinity to the Fibril End as a Crucial Factor. J. Mol. Biol. 2020, 432 (4), 967–977. 10.1016/j.jmb.2019.11.019. 31809698

[ref6] Kyriukha Y. A.; Afitska K.; Kurochka A. S.; Sachan S.; Galkin M.; Yushchenko D. A.; Shvadchak V. V. alpha-Synuclein Dimers as Potent Inhibitors of Fibrillization. J. Med. Chem. 2019, 62 (22), 10342–10351. 10.1021/acs.jmedchem.9b01400. 31625739

[ref7] Serpell L. C.; Berriman J.; Jakes R.; Goedert M.; Crowther R. A. Fiber diffraction of synthetic alpha-synuclein filaments shows amyloid-like cross-beta conformation. Proc. Natl. Acad. Sci. U. S. A 2000, 97 (9), 4897–4902. 10.1073/pnas.97.9.4897. 10781096 PMC18329

[ref8] Chen J.; Malone B.; Llewellyn E.; Grasso M.; Shelton P. M. M.; Olinares P. D. B.; Maruthi K.; Eng E. T.; Vatandaslar H.; Chait B. T.; et al. Structural Basis for Helicase-Polymerase Coupling in the SARS-CoV-2 Replication-Transcription Complex. Cell 2020, 182 (6), 1560–1573e1513. 10.1016/j.cell.2020.07.033. 32783916 PMC7386476

[ref9] Brundin P.; Melki R. Prying into the Prion Hypothesis for Parkinson’s Disease. J. Neurosci. 2017, 37 (41), 9808–9818. 10.1523/JNEUROSCI.1788-16.2017. 29021298 PMC5637113

[ref10] Miraglia F.; Ricci A.; Rota L.; Colla E. Subcellular localization of alpha-synuclein aggregates and their interaction with membranes. Neural Regen. Res. 2018, 13 (7), 1136–1144. 10.4103/1673-5374.235013. 30028312 PMC6065224

[ref11] Haddad F.; Sawalha M.; Khawaja Y.; Najjar A.; Karaman R. Dopamine and Levodopa Prodrugs for the Treatment of Parkinson’s Disease. Molecules 2018, 23 (1), 40 10.3390/molecules23010040. PMC5943940 29295587

[ref12] Carrera I.; Cacabelos R. Current Drugs and Potential Future Neuroprotective Compounds for Parkinson’s Disease. Curr. Neuropharmacol. 2019, 17 (3), 295–306. 10.2174/1570159X17666181127125704. 30479218 PMC6425078

[ref13] Stoker T. B.; Torsney K. M.; Barker R. A. Emerging Treatment Approaches for Parkinson’s Disease. Front. Neurosci. 2018, 12, 693 10.3389/fnins.2018.00693. 30349448 PMC6186796

[ref14] Fields C. R.; Bengoa-Vergniory N.; Wade-Martins R. Targeting Alpha-Synuclein as a Therapy for Parkinson’s Disease. Front. Mol. Neurosci. 2019, 12, 299 10.3389/fnmol.2019.00299. 31866823 PMC6906193

[ref15] Korshavn K. J.; Jang M.; Kwak Y. J.; Kochi A.; Vertuani S.; Bhunia A.; Manfredini S.; Ramamoorthy A.; Lim M. H. Reactivity of Metal-Free and Metal-Associated Amyloid-beta with Glycosylated Polyphenols and Their Esterified Derivatives. Sci. Rep. 2015, 5, 17842 10.1038/srep17842. 26657338 PMC4674742

[ref16] Javed H.; Nagoor Meeran M. F.; Azimullah S.; Adem A.; Sadek B.; Ojha S. K. Plant Extracts and Phytochemicals Targeting alpha-Synuclein Aggregation in Parkinson’s Disease Models. Front. Pharmacol. 2019, 9, 1555 10.3389/fphar.2018.01555. 30941047 PMC6433754

[ref17] Ghanem S. S.; Fayed H. S.; Zhu Q.; Lu J. H.; Vaikath N. N.; Ponraj J.; Mansour S.; El-Agnaf O. M. A. Natural Alkaloid Compounds as Inhibitors for Alpha-Synuclein Seeded Fibril Formation and Toxicity. Molecules 2021, 26 (12), 3736 10.3390/molecules26123736. 34205249 PMC8234408

[ref18] Oliveri V. Toward the discovery and development of effective modulators of α-synuclein amyloid aggregation. Eur. J. Med. Chem. 2019, 167, 10–36. 10.1016/j.ejmech.2019.01.045. 30743095

[ref19] Peña-Díaz S.; Pujols J.; Conde-Giménez M.; Čarija A.; Dalfo E.; García J.; Navarro S.; Pinheiro F.; Santos J.; Salvatella X.; et al. ZPD-2, a Small Compound That Inhibits α-Synuclein Amyloid Aggregation and Its Seeded Polymerization. Front. Mol. Neurosci. 2019, 12, 306 10.3389/fnmol.2019.00306. 31920537 PMC6928008

[ref20] Wagner J.; Ryazanov S.; Leonov A.; Levin J.; Shi S.; Schmidt F.; Prix C.; Pan-Montojo F.; Bertsch U.; Mitteregger-Kretzschmar G.; et al. Anle138b: a novel oligomer modulator for disease-modifying therapy of neurodegenerative diseases such as prion and Parkinson’s disease. Acta Neuropathol. 2013, 125 (6), 795–813. 10.1007/s00401-013-1114-9. 23604588 PMC3661926

[ref21] Pujols J.; Peña-Díaz S.; Lázaro D. F.; Peccati F.; Pinheiro F.; González D.; Carija A.; Navarro S.; Conde-Giménez M.; García J.; et al. Small molecule inhibits α-synuclein aggregation, disrupts amyloid fibrils, and prevents degeneration of dopaminergic neurons.. Proc. Natl. Acad. Sci. U. S. A 2018, 115 (41), 10481–10486. 10.1073/pnas.1804198115. 30249646 PMC6187188

[ref22] Vittorio S.; Adornato I.; Gitto R.; Pena-Diaz S.; Ventura S.; De Luca L. Rational design of small molecules able to inhibit alpha-synuclein amyloid aggregation for the treatment of Parkinson’s disease. J. Enzyme Inhib. Medi. Chem. 2020, 35 (1), 1727–1735. 10.1080/14756366.2020.1816999. PMC7534360 32924648

[ref23] Bayrak H.; Demirbas A.; Demirbas N.; Karaoglu S. A. Synthesis of some new 1,2,4-triazoles starting from isonicotinic acid hydrazide and evaluation of their antimicrobial activities. Eur. J. Med. Chem. 2009, 44 (11), 4362–4366. 10.1016/j.ejmech.2009.05.022. 19647352

[ref24] Sung K.; Lee A. R. Synthesis of [(4,5-Disubstituted-4h-1,2,4-Triazol-3-Yl)Thio]Alkanoic Acids and Their Analogs as Possible Antiinflammatory Agents. J. Heterocyclic Chem. 1992, 29 (5), 1101–1109. 10.1002/jhet.5570290512.

[ref25] Jiang X.; Tang G. Y.; Yang J.; Ding J. C.; Lin H. W.; Xiang X. L. Synthesis of some new acylhydrazone compounds containing the 1,2,4-triazole structure and their neuritogenic activities in Neuro-2a cells. Rsc. Adv. 2020, 10 (32), 18927–18935. 10.1039/D0RA02880K. 35518339 PMC9053900

[ref26] Paterniti I.; Campolo M.; Siracusa R.; Cordaro M.; Di Paola R.; Calabrese V.; Navarra M.; Cuzzocrea S.; Esposito E. Liver X receptors activation, through TO901317 binding, reduces neuroinflammation in Parkinson’s disease. PLoS One 2017, 12 (4), e0174470 10.1371/journal.pone.0174470. 28369131 PMC5378346

[ref27] Crupi R.; Impellizzeri D.; Cordaro M.; Siracusa R.; Casili G.; Evangelista M.; Cuzzocrea S. N-palmitoylethanolamide Prevents Parkinsonian Phenotypes in Aged Mice. Mol. Neurobiol. 2018, 55 (11), 8455–8472. 10.1007/s12035-018-0959-2. 29552727

[ref28] Cordaro M.; Siracusa R.; Crupi R.; Impellizzeri D.; Peritore A. F.; D’Amico R.; Gugliandolo E.; Di Paola R.; Cuzzocrea S. 2-Pentadecyl-2-Oxazoline Reduces Neuroinflammatory Environment in the MPTP Model of Parkinson Disease. Mol. Neurobiol. 2018, 55 (12), 9251–9266. 10.1007/s12035-018-1064-2. 29656363

[ref29] Pujols J.; Pena-Diaz S.; Conde-Gimenez M.; Pinheiro F.; Navarro S.; Sancho J.; Ventura S. High-Throughput Screening Methodology to Identify Alpha-Synuclein Aggregation Inhibitors. Int. J. Mol. Sci. 2017, 18 (3), 478 10.3390/ijms18030478. 28257086 PMC5372494

[ref30] Pena-Diaz S.; Pujols J.; Pinheiro F.; Santos J.; Pallares I.; Navarro S.; Conde-Gimenez M.; Garcia J.; Salvatella X.; Dalfo E.; et al. Inhibition of alpha-Synuclein Aggregation and Mature Fibril Disassembling With a Minimalistic Compound, ZPDm. Front. Bioeng. Biotechnol. 2020, 8, 588947 10.3389/fbioe.2020.588947. 33178678 PMC7597392

