# Supplementary material for: Discovery of Neuroprotective Agents Based on a 5-(4-Pyridinyl)-1,2,4-triazole Scaffold
Source: ACS Chem Neurosci. 2022 Feb 18;13(5):581–6. doi: 10.1021/acschemneuro.1c00849 (PMC9937533; doi:10.1021/acschemneuro.1c00849)
Supplement: Supplementary file 1 — cn1c00849_si_001.pdf [file cn1c00849_si_001.pdf]

Supporting Information

Discovery of neuroprotective agents based on a 5-(4-pyridinyl)-1,2,4-triazole scaffold

Rosaria Gitto[a], Serena Vittorio[a], Federica Bucolo[a], Samuel Peña-Díaz[b,c], Rosalba Siracusa[a], Salvatore Cuzzocrea[a], Salvador Ventura[b,c,d], Rosanna Di Paola[a], Laura De Luca[a*]

[a]*Department of Chemical, Biological, Pharmaceutical and Environmental Sciences, University of Messina, Viale F. Stagno D'Alcontres 31, 98125 Messina, Italy.*
[b]*Institut de Biotecnologia i Biomedicina, Universitat Autonoma de Barcelona, 08193 Bellaterra, Spain.*
[c]*Departament de Bioquimica i Biologia Molecular, Universitat Autonoma de Barcelona, 08193 Bellaterra, Spain.*
[d]*ICREA, Passeig Lluis Companys 23, 08010 Barcelona, Spain.*

Content

1. **Experimental details for compounds 13-15**
2. **Supplementary data of *in vivo* study**

1. Experimental details for compounds 13-15

*3-(Ethylthio)-5-(pyridin-4-yl)-4H-1,2,4-triazol-4-amine **(13)*** CAS Number: 901096-40-0; Yield: 72.5%; m.p.: 125-127°C; ^{1}H-NMR (500 MHz, DMSO-*d_6*): 1.36 (t, J= Hz, 3H, CH_3), 3.16-3.21 (m, 2H; CH_2), 6.21 (bs, 2H, NH_2), 7.99-8.01 (m, 2H, ArH), 8.71-8.72 (m, 2H, ArH); Anal. for ($C_9H_{11}N_5S$): C, 48.85%; H, 5.01%; N, 31.65%; Found: C, 48.70%; H, 5.16%; N, 31.50%

*Methyl 2-((4-amino-5-(pyridin-4-yl)-4H-1,2,4-triazol-3-yl)thio)acetate **(14)*** CAS Number: 150535-95-8; Yield: 38%; m.p.: 190-192 °C; ^{1}H-NMR (500 MHz, DMSO-*d_6*): 3.66 (s, 3H, CH_3), 4.12 (s, 2H; CH_2), 6.31 (bs, 2H, NH_2), 7.96-7.97 (m, 2H, ArH), 8.71-8.72 (m, 2H, ArH); Anal. for ($C_{10}H_{11}N_5O_2S$): C, 45.27%; H, 4.18%; N, 26.40%; Found: C, 45.42%; H, 4.03%; N, 26.55%

*Ethyl 2-((4-amino-5-(pyridin-4-yl)-4H-1,2,4-triazol-3-yl)thio)acetate **(15)*** CAS Number: 39875-99-5; Yield: 55%; m.p.: 180-82 °C; ^{1}H-NMR (500 MHz, DMSO-*d_6*): 1.18 (t, J= 7.05 Hz, 3H, CH_3), 4.10 (s, 2H; CH_2), 4.11 (q, J= 7.05 Hz, 2H, CH_2), 6.34 (bs, 2H, NH_2), 7.96-7.97 (m, 2H, ArH), 8.70-8.71

(m, 2H, ArH); Anal. for ($C_{11}H_{13}N_5O_2S$): C, 47.30%; H, 4.69%; N, 25.07%; Found: C, 47.15%; H, 4.84%; N, 24.92%.

2. Supplementary data of *in vivo* study

2.1 Methods

Animals. C57/BL6 mice (male 25–30 g; Envigo, Milan, Italy) were housed in an organized environment and equipped with standard rodent food and water. Mice were housed in cages in a room kept at 22 ± 1°C with a 12-h light, 12-h dark cycle. Mice were acclimatized to their habitat for 1 week and they had ad libitum access to tap water and rodent standard food. The University of Messina Review Board for animal care (OPBA) approved the study. All animal experiments agree with the new Italian regulations (D.Lgs 2014/26), EU regulations (EU Directive 2010/63), and the ARRIVE guidelines.

MPTP-Induced PD and Treatments. Eight-week-old male C57/BL6 mice were treated with MPTP or saline. For MPTP intoxication, mice received 4 intraperitoneal (i.p.). injections of MPTP (20 mg/kg; Sigma, Milan Italy) in saline solution at 2 hours intervals in 1 day: total dose per animal was 80 mg/kg. For Seril-18 treatment (10 mg/kg, orally), mice received this compound starting 24 hours after the first MPTP administration and continuing through 7 additional days after the last administration of MPTP. Sham animals received vehicle only (saline). Eight days after MPTP injection, mice were sacrificed by decapitation. Brains were dissected out, and midbrains were isolated and processed. The dose of MPTP (20 mg/kg) used was based on previous *in vivo* studies.[1, 2]

Behavioral Testing. Behavioral evaluations on all mice were made 1 day prior to, and 8 days after, MPTP injection:

Rotarod Test (RT). Behavioral valuations on each mouse were made 1 day prior to, and 8 days after, MPTP injection. Motor performance was measured with a rotary rod apparatus using a protocol similar to that described.[3] Briefly, the mice were placed on the rotating rod and the time until they fell off was noted. This was repeated (with a rest period that increased by 5 seconds with each fall) until the total time on the rod for the Sham group was 5 minutes. Both the total time spent on the rotating rod and the total number of falls for each mouse was documented.

Catalepsy Test (CT). Catalepsy, defined as a reduced ability to initiate movement and correct posture, was measured with the bar test 8 days after the MPTP injection. To assess catalepsy, the mice were positioned so that the hind paws were on the bench and the forelimbs rested on a horizontal bar 1 cm in diameter and 4 cm high. The time the mice assumed this position was recorded up to a maximum

of 180 seconds. Mice were termed cataleptic if they held this position for 30 seconds or more. The animals were returned to their home cage after each trance measurement.[4]

Pole test (PT). The PT was performed as previously described.[5] Briefly, the test consists of a 50-cm high, gauze-taped pole (1 cm in diameter). Animals are placed with their head upwards right below the top. Two parameters were assessed: time until the animal turned by 180° and time until the animal descended to the floor.

Western blot analysis for Dopamine Transporter (DAT). Western blot analysis was performed on brains of 5 mice for each group with the protocol that we previously described.[6] The level of DAT was quantified in cytosolic. The specific primary antibody that was incubated at 4 °C overnight was anti-DAT (Santa Cruz Biotechnology, 1:500, 65G10 sc-32258, Dallas, TX, USA). Protein lysates were also incubated with a β-actin antibody (1:5000; Santa Cruz Biotechnology, C4 sc-47778, Dallas, TX, USA), in order to verify that all samples had been loaded in equal quantities. The signals were captured with BIORAD ChemiDocTM XRS + software thanks to the use of a reagent that emits chemiluminescence (Super Signal West Pico, Pierce chemiluminescent substrate). The relative expression of the bands was subsequently normalized to the β-actin levels. Image analysis was performed using Image Quant TL software, v2003.[7]

2.2 Results

To investigate the relationship between the degeneration of dopaminergic neurons, MPTP-induced, and the recovery processes, we analyzed the motor activity 1 day prior and 8 days after the MPTP induction. The data at time point 0 are not shown as no significant differences between the different groups were observed. By Rotarod Test (RT), mice showed a significant motor disorder as indicated by a reduce in time spent on the apparatus and by a greater number of falls. Treatment with ester **15** significantly reduced this motor dysfunction (Figure S1A). Moreover, the MPTP produced an important cataleptic effect in animals, above all at 8 days after MPTP injection, when the mice exhibited a significant increase in cataleptic symptoms. Importantly, the daily compound **15** administration significantly reduced the catalepsy duration induced by MPTP (Figure S1B). In the Pole Test (PT) “total time” and “time to turn” significantly increased after MPTP injection compared with the Sham group. Compound **15** treatment at 10 mg/kg significantly reduced “total time” and “time to turn” suggesting that **15** prevents MPTP-induced bradykinesia (Figure S1C, C’).

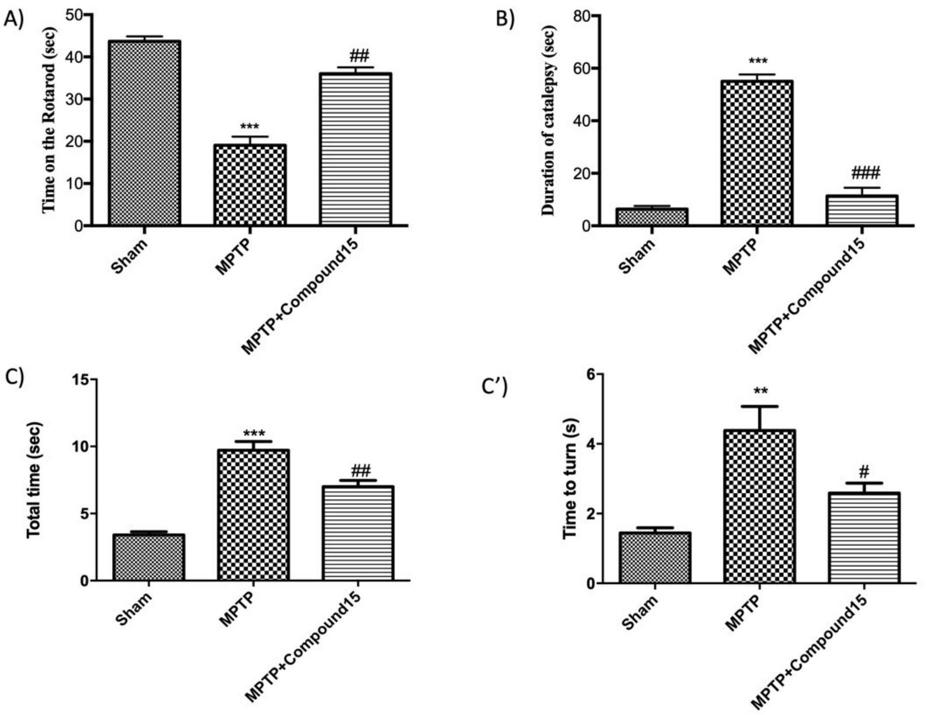

Figure S1. Effect of compound **15** on behavioral impairments induced by MPTP intoxication. (**A**) Motor function was assessed using a Rotarod apparatus. At 8 days, mice exhibited a significant motor dysfunction as indicated by a decrease in time spent on the Rotarod. Compound **15** treatment blunted the motor dysfunction in mice. Values are mean ± SEM (**N** = 5 per group). *****p** < 0.001 vs Sham; ##**p** < 0.01 vs MPTP. (**B**) Catalepsy was evaluated according to the standard bar hanging procedure; this motor test showed that **15** treatment reduced behavioral impairment induced by MPTP. *****p** < 0.001 vs Sham; ###**p** < 0.001 vs MPTP. (**C, C'**) Motor function was assessed using a Pole test. At 8 days, mice exhibited a significant motor dysfunction as indicated by an increase in "Time to turn" and "Total time" spent to descend to the floor following injection of MPTP compared with the Sham group. Compound **15** administration notably reduced "Total time" and "Time to turn". *****p** < 0.001 vs Sham; ****p** < 0.01 vs Sham; ##**p** < 0.01 vs MPTP; #**p** < 0.05 vs MPTP.

With Western blot analysis we observed the level of DAT. Briefly, expression of DAT significantly decreased after MPTP injection compared to the Sham group. Treatment with **15** significantly increased DAT expression (Figure S2A, A').

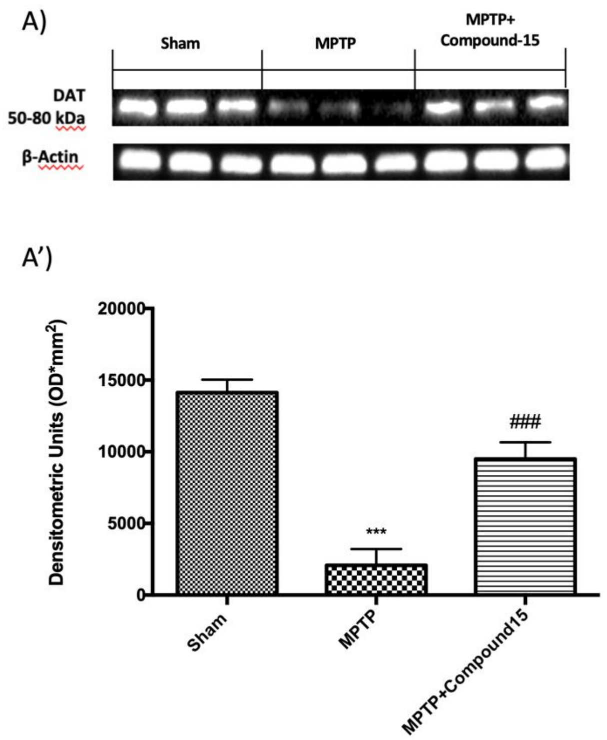

Figure S2. Effect of **15** treatment on DAT expression after MPTP-intoxication. Western blot analysis on midbrain region revealed a significant decrease in DAT in the MPTP group compared to Sham animals. Compound **15** treatment significantly reduced the decrease in this protein (**A**). The data are expressed as the mean SEM from n = 5 mice/group. (**A'**) ***$\mathbf{p} < 0.001$ vs Sham; ###$\mathbf{p} < 0.001$ vs MPTP.

References

(1) Crupi, R.; Impellizzeri, D.; Cordaro, M.; Siracusa, R.; Casili, G.; Evangelista, M.; Cuzzocrea, S., N-palmitoylethanolamide Prevents Parkinsonian Phenotypes in Aged Mice. *Mol Neurobiol* **2018,** *55* (11), 8455-8472. DOI: 10.1007/s12035-018-0959-2

(2) Paterniti, I.; Campolo, M.; Siracusa, R.; Cordaro, M.; Di Paola, R.; Calabrese, V.; Navarra, M.; Cuzzocrea, S.; Esposito, E., Liver X receptors activation, through TO901317 binding, reduces neuroinflammation in Parkinson's disease. *PLoS One* **2017,** *12* (4), e0174470. DOI: 10.1371/journal.pone.0174470

(3) Campolo, M.; Paterniti, I.; Siracusa, R.; Filippone, A.; Esposito, E.; Cuzzocrea, S., TLR4 absence reduces neuroinflammation and inflammasome activation in Parkinson's diseases in vivo model. *Brain Behav Immun* **2019,** *76*, 236-247. DOI: 10.1016/j.bbi.2018.12.003

(4) Araki, T.; Kumagai, T.; Tanaka, K.; Matsubara, M.; Kato, H.; Itoyama, Y.; Imai, Y., Neuroprotective effect of riluzole in MPTP-treated mice. *Brain Res* **2001,** *918* (1-2), 176-81. DOI: 10.1016/s0006-8993(01)02944-4

(5) Sedelis, M.; Schwarting, R. K.; Huston, J. P., Behavioral phenotyping of the MPTP mouse model of Parkinson's disease. *Behav Brain Res* **2001,** *125* (1-2), 109-25. DOI: 10.1016/s0166-4328(01)00309-6

(6) Siracusa, R.; Paterniti, I.; Cordaro, M.; Crupi, R.; Bruschetta, G.; Campolo, M.; Cuzzocrea, S.; Esposito, E., Neuroprotective Effects of Temsirolimus in Animal Models of Parkinson's Disease. *Mol Neurobiol* **2018,** *55* (3), 2403-2419. DOI: 10.1007/s12035-017-0496-4

(7) Siracusa, R.; Scuto, M.; Fusco, R.; Trovato, A.; Ontario, M. L.; Crea, R.; Di Paola, R.; Cuzzocrea, S.; Calabrese, V., Anti-inflammatory and Anti-oxidant Activity of Hidrox((R)) in

Rotenone-Induced Parkinson's Disease in Mice. *Antioxidants (Basel)* **2020,** *9* (9). DOI: 10.3390/antiox9090824
